# Strengthening cross-regional networks: evaluation of Indonesia's national Early Warning Alert and Response System by field epidemiology training programmes from Indonesia and Japan, 2023

**DOI:** 10.5365/wpsar.2025.16.3.1243

**Published:** 2025-09-02

**Authors:** Keiko Tsukada, Irma Gusmi Ratih, Shingo Nishiki, Munehisa Fukusumi, Atik Choirul Hidajah, Fransisca Susilastuti, Anak Agung Sagung Sawitri, I Wayan Sugihana Aradea, Tri Yunis Miko Wahyono, Helda,, Dwi Sutiningsih, Nissa Kusariana, Bayu Satria Wiratama, Sugiarto,, Indra Dwinata, Oderna,, Isamu Kuboki, Rizki Dinar Winiar, Risky Ayunni, Nur Assyifa Daiyah Fillah, Eka Muhiriyah, Yulia Zubir, Emita Ajis, Cepi Irawansyah, Crysti Mei Manik, Firda Aisya, Husni,, Orina Melisa Nasution, Tomimasa Sunagawa, Triya Novita Dinihari

**Affiliations:** a Center for Field Epidemic Intelligence, Research and Professional Development, National Institute of Infectious Diseases, Tokyo, Japan.; b Directorate of Surveillance and Health Quarantine, Ministry of Health, Jakarta, Indonesia.; c Project for Strengthening Capacity for Early Warning and Response to Infectious Diseases, Japan International Cooperation Agency, Jakarta, Indonesia.; d Field Epidemiology Training Program Indonesia, Ministry of Health, Jakarta, Indonesia.; e Department of Epidemiology, Biostatistics, Population and Health Promotion, Universitas Airlangga, Surabaya, Indonesia.; f Public Health Laboratory, Surabaya, Indonesia.; g Department of Public Health and Preventive Medicine, Faculty of Medicine, Udayana University, Denpasar, Bali, Indonesia.; h Bali Provincial Health Office, Bali, Indonesia.; i Department of Epidemiology, Faculty of Public Health, University of Indonesia, Depok, Indonesia.; j Department of Epidemiology and Tropical Diseases, Faculty of Public Health, University of Diponegoro, Semarang, Central Java, Indonesia.; k Department of Biostatistics, Epidemiology and Population Health, Faculty of Medicine, Public Health and Nursing, Universitas Gadjah Mada, Yogyakarta, Indonesia.; l Kulon Progo District Health Office, Yogyakarta, Indonesia.; m Department of Epidemiology, Faculty of Public Health, Hasanuddin University, Makassar, Indonesia.; n Soppeng District Health Office, South Sulawesi, Indonesia.; o Public Health Emergency Operations Center, Ministry of Health, Jakarta, Indonesia.

The field epidemiology training programmes (FETPs) in Indonesia and Japan conducted a joint evaluation of surveillance by Indonesia's national Early Warning Alert and Response System (EWARS) in 2023. This effort was carried out under the supervision of the Indonesian Ministry of Health and in collaboration with the Japan International Cooperation Agency (JICA) EWARS Project. This collaborative activity underscored the importance of cooperation among FETPs to facilitate knowledge-sharing and network-strengthening. This report provides an overview of the joint project, highlighting key insights from the activity.

As of 2023, 98 FETPs have trained public health staff across more than 200 countries and territories. (*1*) These programmes are primarily designed to develop in-country human resources to strengthen public health systems. Recent evidence highlights the contributions of FETP trainees and alumni in enhancing the workforce’s capacity for epidemiology and improving public health emergency infrastructure, particularly through their involvement in COVID-19 preparedness and response activities in different countries. (*2*) The COVID-19 pandemic further underscored the necessity of strengthening networks across countries and regions to ensure global health security.

While the content of the FETPs is tailored to the specific circumstances of each country, most are modelled on the Epidemic Intelligence Service, established in 1951 by the United States Centers for Disease Control and Prevention (CDC). (*3*) Additionally, the Training Programs in Epidemiology and Public Health Interventions Network (also known as TEPHINET), the global network of FETPs, set standards for activities and deliverables within the curriculum, supporting the delivery of quality training. (*4*) Among the programme’s components, the evaluation of surveillance activities or systems is one of the core epidemiological practices. The standard guidelines for this type of evaluation, developed by the CDC, have been adopted by various FETPs, (*4*-*6*) including FETP Indonesia and FETP Japan. Using this shared methodology, these two FETPs collaboratively evaluated Indonesia's EWARS.

EWARS, a web-based syndromic surveillance system established in Indonesia in 2009, aims to facilitate the early detection of public health events to enable a prompt response. (*7*, *8*) It monitors 24 types of diseases and syndromes through indicator-based surveillance, and issues weekly reports. The system operates across four hierarchical levels, from the national level down to the provincial, district and subdistrict levels, with Puskesmas (public health centres) serving as the primary reporting units at the subdistrict level. Aggregated data are transmitted weekly, primarily from reporting units to the national server via short message service (also known as SMS). (*7*, *8*) The EWARS database, administered by the Ministry of Health, includes aggregated weekly case counts sorted by disease type and reporting source for indicator-based surveillance.

Twelve alumni and facilitators from FETP Indonesia, representing six public universities that offer the Programme, participated in the collaborative project alongside four alumni from FETP Japan, who were dispatched by Japan’s National Institute of Infectious Diseases. Necessary preparations were made in accordance with the CDC’s guidelines for evaluating public health surveillance systems. (*5*) First, a concept note was created by the participants to clarify the background, purpose, assessment methods and target diseases for the evaluation. Dengue fever and measles were selected as the evaluation targets, and the criteria used in the assessment are outlined in **Table 1**. Second, standardized questionnaires for data collection were collaboratively developed for each disease in both English and Indonesian. These questionnaires included attribute-based questions for qualitative analysis and indicators for quantitative analysis. Data were collected from face-to-face interviews with relevant surveillance officers and from EWARS and other surveillance databases. Informed consent was obtained from all respondents, and no personally identifiable information was collected.

Evaluation of the surveillance system was conducted from 6 November to 15 December 2023 in all three target provinces of the JICA EWARS Project (Banten, East Kalimantan and South Sulawesi), with each province assessed for 2 weeks. During the first week, the joint teams visited one provincial health office, two district health offices and four Puskesmas. They used the questionnaire to conduct interviews with surveillance officers, primarily in Indonesian, facilitated by the FETP Indonesia project members. A wrap-up session was held on the final day of the first week to share findings and challenges among all team members. During the second week, project members collaborated online to analyse quantitative and qualitative data and summarize the results. On the final day of the project, the findings, along with joint recommendations, were presented at an online seminar attended by more than 70 participants from national and local government agencies. Additionally, the joint evaluation activity was showcased at the 11th National Scientific Conference on Epidemiology in Indonesia in August 2024.

This collaboration, which used the standardized evaluation framework developed by the US CDC and shared by both FETPs, provided valuable insights, especially for the non-host FETP. FETP Japan alumni gained a better understanding of common reporting challenges related to notifiable syndromes in both Indonesia’s EWARS and Japan’s National Epidemiological Surveillance of Infectious Diseases (NESID) system. For instance, while laboratory confirmation for dengue fever is not required by Indonesia’s EWARS (**Table 1**), some Puskesmas reported dengue cases that were positive only by rapid diagnostic test (RDT). This misunderstanding of the case definition may reflect the influence on staff members’ reporting behaviour of the widespread availability of RDTs for dengue at the evaluated sites. A similar pattern has been observed in Japan’s NESID for surveillance of infuenza-like illness, in which cases diagnosed with RDTs predominate, along with some clinically diagnosed cases. (*9*, *10*) As a result, mutual understanding through the joint evaluation fostered a robust relationship between FETPs in Indonesia and Japan, paving the way for future collaboration.

Implementing the joint evaluation revealed several challenges. First, bidirectional translation between English and Indonesian, particularly during the development of questionnaires and interviews, posed difficulties in terms of maintaining the accuracy of information. However, close communication among project members helped address this issue. Second, a knowledge gap emerged due to the different backgrounds of alumni from the two FETPs. However, involving individuals familiar with the surveillance systems of both countries facilitated smoother discussions and operations, thereby enhancing the joint evaluation. Specifically, JICA project members played a crucial role in overcoming this challenge. Third, the joint evaluation of the surveillance system required participants to possess a certain level of skills and knowledge, setting high expectations for FETP trainees and resulting in all participants being either alumni or facilitators. However, in the future, once a project’s framework and methodology are clearly established, FETP trainees with relevant domestic experience could also participate in joint projects with technical support from senior FETP alumni. Additionally, participation in overseas projects has been reported to help trainees develop interpersonal skills in international settings, and these are often challenging to acquire through domestic training. (*11*) Lastly, securing FETP budgets for international networking is essential for promoting collaborative activities.

This report shares the experiences of a joint evaluation of the Indonesian EWARS, which utilizes an evaluation framework prevalent among FETPs worldwide. This model can be applied to FETPs in other countries to strengthen the global network through collaborative activities. Therefore, this experience could serve as a meaningful milestone and model, particularly given the growing need for enhanced global networking to improve global health security.
